# Supplementary material for: Bovine Serum Albumin-Based Nanoparticles: Preparation, Characterization, and Antioxidant Activity Enhancement of Three Main Curcuminoids from Curcuma longa
Source: Molecules. 2022 Apr 25;27(9):2758. doi: 10.3390/molecules27092758 (PMC9106055; doi:10.3390/molecules27092758)
Supplement: Supplementary file 1 [file molecules-27-02758-s001.zip › molecules-1639556-supplementary.pdf]

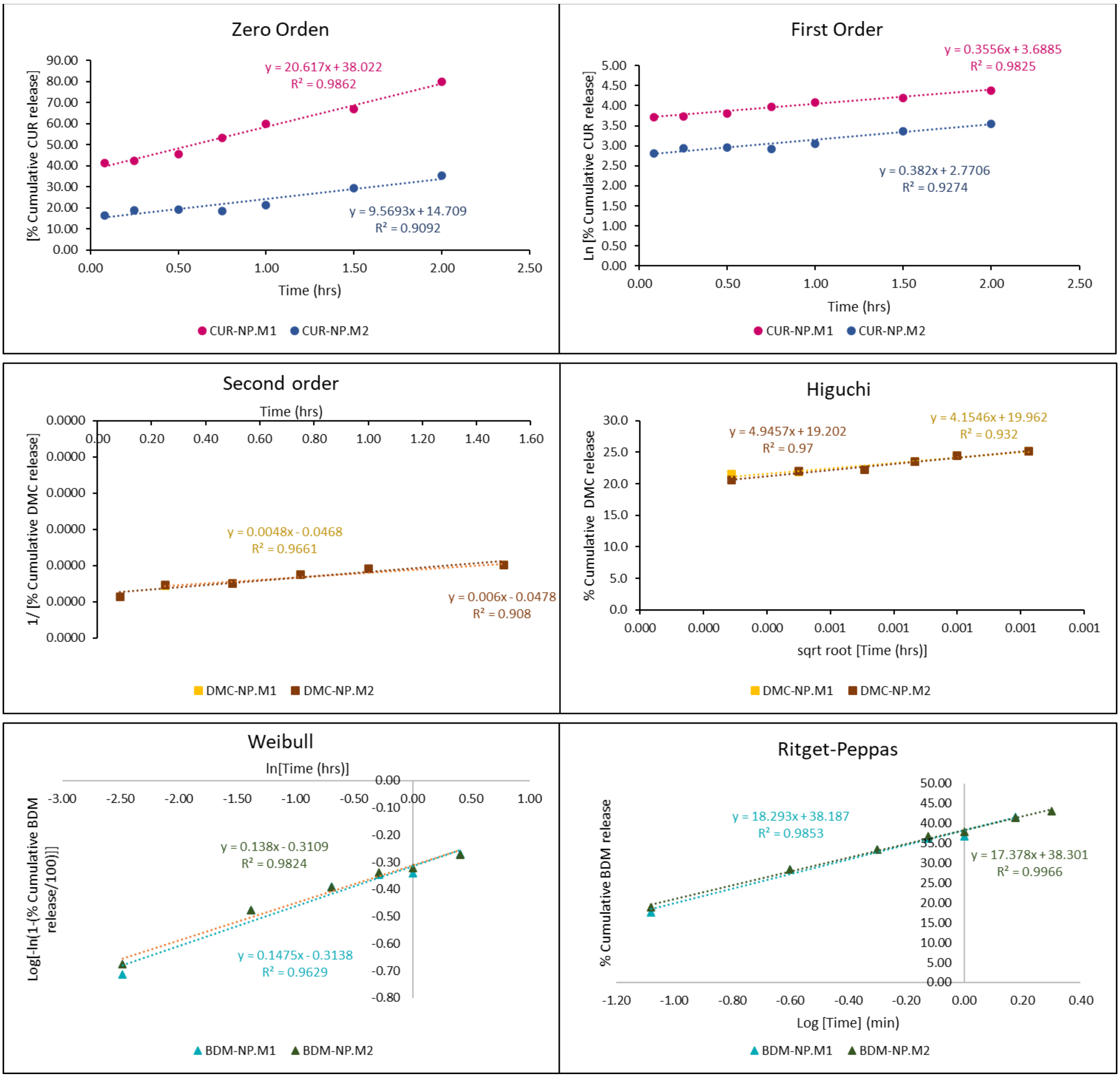

Figure S1. Fitting models of best release kinetics in the experimental data for CUR-NP, DMC-NP, and BDM-NP in M_1 and M_2 in six different kinetic models: zero order, first order, second order, Ritget-Peppas, Higuchi and Weibull
